# RE-HEPATECTOMY MEANS MORE MORBIDITY? A MULTICENTRIC ANALYSIS

**DOI:** 10.1590/0102-672020210002e1647

**Published:** 2022-06-17

**Authors:** Luiza BASILIO, Klaus STEINBRÜCK, Reinaldo FERNANDES, Marcelo D’OLIVEIRA, Renato CANO, Hanna VASCONCELOS, Daniel BARBOSA, Marcelo ENNE

**Affiliations:** 1 Federal Hospital of Ipanema, Hepatobiliary Surgery Service, Rio de Janeiro, RJ, Brazil;; 2 Hepatobiliary Multidisciplinary Service, Rio de Janeiro, RJ, Brazil;; 3 Federal Hospital of Bonsucesso, Hepatobiliary Surgery Service, Rio de Janeiro, RJ, Brazil;; 4 Servidores Federal Hospital, Hepatobiliary Surgery Service, Rio de Janeiro, RJ, Brazil

**Keywords:** Liver, Hepatectomy, Morbidity, Mortality, Fígado, Hepatectomia, Morbidade, Mortalidade

## Abstract

Colorectal cancer generally metastasizes to the liver. Surgical resection of liver metastasis, which is associated with systemic chemotherapy, is potentially curative, but many patients will present recurrence. In selected patients, repeated hepatectomy is feasible and improves overall survival.

**AIM:**

This study aimed to analyze patients with colorectal liver metastasis (CRLM) submitted to hepatectomy in three centers from Rio de Janeiro, over the past 10 years, by comparing the morbidity of first hepatectomy and re-hepatectomy.

**METHODS:**

From June 2009 to July 2020, 192 patients with CRLM underwent liver resection with curative intent in three hospitals from Rio de Janeiro Federal Health System. The data from patients, surgeries, and outcomes were collected from a prospectively maintained database. Patients submitted to first and re-hepatectomies were classified as Group 1 and Group 2, respectively. Data from groups were compared and value of p<0.05 was considered significant.

**RESULTS:**

Among 192 patients, 16 were excluded. Of the remaining 176 patients, 148 were included in Group 1 and 28 were included in Group 2. Fifty-five (37.2%) patients in Group 1 and 13 (46.5%) in Group 2 presented postoperative complications. Comparing Groups 1 and 2, we found no statistical difference between the cases of postoperative complications (p=0.834), number of minor (p=0.266) or major (p=0.695) complications, and deaths (p=0.407).

**CONCLUSIONS:**

No differences were recorded in morbidity or mortality between patients submitted to first and re-hepatectomies for CRLM, which reinforces that re-hepatectomy can be performed with outcomes comparable to first hepatectomy.

## RESUMO - RACIONAL

O câncer colorretal geralmente metastatiza para o fígado. Hepatectomia associada à quimioterapia sistêmica é potencialmente curativa para metástases hepáticas colorretais, entretanto, muitos pacientes apresentarão recidiva após a cirurgia. Em casos selecionados, a re-hepatectomia é viável, com relatos de melhora na sobrevida global.

**OBJETIVO:**

Analisar pacientes com metástase hepática colorretal operados em três centros do Rio de Janeiro, nos últimos 10 anos, comparando as morbidades da primeira hepatectomia e da re-hepectomia.

**MÉTODOS:**

De junho de 2009 a julho de 2020, 192 pacientes com metástase hepática colorretal foram submetidos à hepatectomia em três hospitais do Rio de Janeiro. Os dados dos pacientes, cirurgias e desfechos foram coletados de um banco de dados mantido prospectivamente. Pacientes submetidos à primeira hepatectomia e re-hepatectomia foram classificados como Grupo 1 e Grupo 2, respectivamente. Os dados dos grupos foram comparados e o valor de p<0,05 foi considerado significativo.

**RESULTADOS:**

Dentre 192 pacientes, dezesseis foram excluídos. Dos 176 pacientes restantes, 148 e 28 foram incluídos dos Grupos 1 e 2, respectivamente. Cinquenta e cinco (37,2%) pacientes no Grupo 1 e treze (46,5%) no Grupo 2 apresentaram complicações pós-operatórias. Comparando os Grupos 1 e 2, não foi observada diferença estatística entre o número de pacientes com complicações pós-operatórias (p = 0,834), complicações menores (p = 0,266) ou maiores (p = 0,695) e óbitos (p = 0,407).

**CONCLUSÕES:**

Não foram registradas diferenças na morbidade ou mortalidade entre os pacientes submetidos à primeira ou à re-hepatectomia em pacientes com metástase hepática colorretal, o que sustenta que a re-hepatectomia pode ser realizada com resultados comparáveis à primeira hepatectomia.

## INTRODUCTION

Colorectal cancer generally metastasizes to the liver and/or lungs. At the time of diagnosis, approximately 25% of patients have metastasis and nearly 30% will develop it during the course of the disease^2^ ^,^ ^15^ ^,^ ^16^. Multidisciplinary treatment, i.e., matching surgical resection and systemic chemotherapy is potentially curative, with a 5-year overall survival rate of 40-60%^6^ ^,^ ^18^ ^,^ ^25^. Nevertheless, recurrence is common, around 50% in the first 2 years after resection, and the liver is the principal site^8^ ^,^ ^9^ ^,^ ^28^. In selected patients, repeated liver resection is feasible and improves the overall survival^17^ ^,^ ^20^ ^,^ ^27^. The benefits and outcomes after repeated liver resection and selection of patients still need discussion.

This study aimed to evaluate patients with colorectal liver metastasis (CRLM) treated with surgery in three centers from Rio de Janeiro, over the past 10 years, by focusing on the safety and outcomes of hepatectomies and comparing the morbidity of first hepatectomy and re-hepatectomy.

## METHODS

From June 2009 to July 2020, 192 patients with CRLM underwent liver resection in three hospitals from Rio de Janeiro’s Federal Health System, namely Ipanema Federal Hospital, Bonsucesso Federal Hospital, and Servidores Federal Hospital. The data from patients, surgeries, and outcomes were collected from a prospectively maintained database. Patients submitted to first and re-hepatectomies were classified as Group 1 and Group 2, respectively.

Preoperatively, all patients were submitted to laboratory tests (including CEA and CA19.9 levels), nutritional evaluation, colonoscopy, and enhanced CT scan (thorax and abdomen). Magnetic resonance imaging (MRI) was performed whenever it was possible. All cases were discussed in a multidisciplinary meeting, and hepatic resection was indicated with curative intent.

Platinum-based chemotherapy was offered to all patients, either preoperatively or postoperatively, according to the multidisciplinary evaluation of each case.

Majority of surgeries were performed by laparotomy with bilateral subcostal or J-shape incisions, depending on patient and tumor features and senior surgeon’s choice. More recently, laparoscopy was used for selected patients with favorable nodule position (left lateral or anterior segments). Resection of more than three segments was considered a major resection. Liver resections were classified according to the Brisbane nomenclature system^23^.

Intraoperative ultrasonography was done routinely to define resection margins. Hepatectomies were performed using an ultrasonic dissector. The Pringle maneuver and the energy devices (monopolar and bipolar cauterizers, vessel sealing device) were used to diminish blood loss. To avoid biliary complications, biliary leakage test^11^ was performed, whenever possible.

Postoperative epidural analgesia was given regularly for all patients submitted to open surgery approach. Subcutaneous low-molecular-weight heparin was initiated on the first postoperative day and maintained until deambulation. Postoperative complications were stratified using the modified Clavien-Dindo scoring system^10^. Grades 1 and 2 were considered minor complications and grades 3 and 4 were considered major complications. Among the patients with more than one complication, only the most severe one was considered.

Data comparison was done using Fisher’s exact test for categorical numbers and Student’s t-test for continuous variables. For statistical significance, p<0.05 was considered significant.

This research was approved by the Ethics Committee of the Institution under the number 0024/2020.

## RESULTS

Among the 192 patients analyzed, 16 were excluded because they were submitted to second-stage hepatectomy and ALLPS procedure or because of lack of data. Of the remaining 176 patients, 148 underwent first hepatectomy (Group 1) and 28 underwent re-hepatectomy (Group 2).

The median age of the Group 1 was 58.22±10.62 years (range 23-81 years), and 62 (41.9%) were females and 86 (58.1%) were males. The majority of patients were classified as those who had ASA 2 (86 patients - 58.1%) and those who had tumors ranging from 3 to 5 cm (64 patients - 43.2%). Major resection was performed in 31 (21%) patients. Median ICU stay and hospitalization time were 2.45±1.95 and 7.28±6.39 days, respectively.

The median age of Group 2 was 54.89±8.80 years (range 36-78 years), and 16 (57.1%) patients were females and 12 (42.8%) were males. Fourteen (50%) patients had tumor with <3 cm in size, and the majority was classified as ASA 2 (17 patients - 60.7%). Major resection was made in 4 (14.3%) patients. Median ICU stay and hospitalization time in Group 2 were 2.29±0.94 and 5.96±1.97 days, respectively. Table 1 shows patients’ demographic data.

**Table 1 -:** Patients’ demographic data.

		Group 1 (N=148)	Group 2 (N=28)	p-value
		N (%)	N (%)	
Gender	Female	62 (41.9)	16 (57.1)	0.151
	Male	86 (58.1)	12 (42.8)	
Age (years)		58.22±10.62	54.89±8.80	0.121
Tumor size	<3 cm	53 (35.8)	14 (50.0)	0.202
	3-5 cm	64 (43.2)	11 (39.3)	0.835
	5-10 cm	23 (15.5)	2 (7.14)	0.376
	>10 cm	3 (2.0)	1 (3.6)	0.503
ASA score	ASA 1	47 (31.7)	10 (35.7)	0.666
	ASA 2	86 (58.1)	17 (60.7)	0.837
	ASA 3	14 (9.4)	1 (3.6)	0.471
	ASA 4	1 (0.7)	-	1.000
Resection	Minor	117 (79)	24 (85.7)	0.606
	Major	31 (21)	4 (14.3)	
Type resection	Non-anatomical	79 (53)	14 (50)	0.837
	Anatomical	53 (36)	14 (50)	
	Both	16 (11)	-	
Blood transfusion		9 (6.0)	1 (3.5)	1.000
ICU time (days)		2.45±1.95	2.29±0.94	0.657
Hospitalization time (days)		7.28±6.39	5.96±1.97	0.281

Sixty-eight patients experienced postoperative complications, i.e., 55 (37.2%) in Group 1 and 13 (46.5%) in Group 2 (Table 2). The most prevalent complications were related to gastrointestinal disorders, such as nausea, vomiting, gastroparesis, and paralytic ileus, which were observed in 22 patients. Nine patients developed biliary complications. Four patients needed to be reoperated. Two patients presented hepatic dysfunction and one developed multiorgan failure, caused by small bowel perforation and peritonitis. There were three deaths: one due to gas embolism, one due to HIV associated with brain tumor hemorrhage, and the last due to severe peritonitis, associated with biliary fistula.

**Table 2 -:** Complications of Group 1 and Group 2.

	Group 1 (N=148)	Group 2 (N=28)
	N (%)	N (%)
Nausea/vomiting	10 (6.7)	3 (10.7)
Gastroparesis/ileum	8 (5.4)	1 (3.6)
Wound infection	5 (3.4)	-
Biliary leak	5 (3.4)	3 (10.7)
Wound dehiscence	3 (2.1)	-
Other wound complications	4 (2.7)	-
Biloma	4 (2.7)	1 (3.6)
Fever	1 (0.7)	1 (3.6)
Hepatic dysfunction	2 (1.4)	-
Pulmonary congestion	-	1 (3.6)
Thrombosis	-	1 (3.6)
Blood transfusion	2 (1.4)	-
Anemia	2 (1.4)	-
Hyperglycemia	1 (0.7)	-
Allergic reaction	1 (0.7)	-
Lipothymia	1 (0.7)	-
Urinary infection	1 (0.7)	-
Headache	1 (0.7)	-
Pneumothorax	1 (0.7)	-
Chylous ascites	1 (0.7)	-
Death	2 (1.4)	1 (3.6)
Total	55 (37.2)	12 (42.8)

Forty-two (28.2%) complications in Group 1 were considered minor, 11 (7.4%) were considered major, and two (1.4%) deaths were observed. In Group 2, 11 (39.3%) complications were considered minor, only one (3.6%) was considered major, and one (3.6%) death was observed (Table 3). Comparing Groups 1 and 2, we found no statistical difference between the number of patients with postoperative complications (p=0.834), number of minor (p=0.266) or major (p=0.695) grade complications, and number of deaths (p=0.407).

**Table 3 -:** Patient’s complications according to Clavien-Dindo classification.

Clavien-Dindo	Group 1 (N=148)	Group 2 (N=28)	p-value
	N (%)	N (%)	
Grade 1	21 (14.1)	7 (25.0)	0.266
Grade 2	21 (14.1)	4 (14.3)	
Grade 3A	6 (4.0)	1 (3.6)	0.695
Grade 3B	3 (2.0)	-	
Grade 4A	1 (0.7)	-	
Grade 4B	1 (0.7)	-	
Grade 5	2 (1.4)	1 (3.6)	0.407
Total	55 (37.2)	13 (46.5)	0.834

## DISCUSSION

Prognosis of patients with colorectal cancer is strongly linked to liver metastasis treatment^5^. Liver is the most common recurrence site, and the multidisciplinary evaluation is important to select benefited patients and the best treatment option^14^. Surgery associated with chemotherapy improves the long-term survival for patients with CRLM recurrence^6^ ^,^ ^18^ ^,^ ^25^; however, morbidity related to hepatectomy is still a significant issue, especially in patients submitted to repeated hepatectomies^4^ ^,^ ^12^ ^,^ ^22^.

Repeated liver resection may be challenging by a combination of reasons, such as adhesions and modifications in the anatomy caused from prior surgery, as well as chemotherapy-induced liver injury^1^. Some initial series have highlighted these factors as responsible for the increased morbimortality associated with such resections^3^ ^,^ ^13^. These results, however, were not observed in more recent studies, which demonstrated no difference in morbimortality between first and re-hepatectomy for CRLM^4^ ^,^ ^12^ ^,^ ^17^ ^,^ ^24^.

Fukami et al.^12^ demonstrated that accumulated experience may play a role to diminish morbidity after re-hepatectomy. In contrast, even high-level centers tend to present higher morbidity after re-hepatectomy, when compared to first hepatectomy for CRLM (p=0.069), as reported by Wicherts et al.^27^ Moreover, in the same report, hepatic complications after re-hepatectomy were more often classified as major complications (p=0.150). This could be explained by the high number of patients with multiple cycles of chemotherapy and submitted to second, third, and even fourth hepatectomies. In the present study, we also observed similar morbidity rates between the first and re-hepatectomy groups (37.2% and 46.5%, respectively; p=0.834), where the majority were classified as having minor complications, in accordance with the literature.^17^ ^,^ ^24^ ^,^ ^27^ We also observed that the first hepatectomy group was more prone to present major grade complications (7.4% vs. 3.6%, p=0.695).

Considering the type of complication, gastrointestinal motility disorders were the most prevalent, affecting 22 patients, i.e., 18 (12.1%) in Group 1 and 4 (14.3%) in Group 2. There is always a concern raised when major hepatectomy is performed, especially when a large raw liver cut surface is present. Biliary leak-related complications, such as biliary fistula or biloma, occurred in 14 patients, i.e., 9 (7.0%) from Group 1 and 5 (17.8%) from Group 2. These results are similar to previous reports.^4^ ^,^ ^27^ Even though there was no statistical difference between both groups (p=0.506), the re-hepatectomy group had a greater tendency to develop biliary leak complications. This could be explained by the difficulties to identify the cystic duct and perform the bile leak test^11^ in patients formerly submitted to cholecystectomy - commonly executed during first hepatectomy. Most of the observed biliary complications could be considered benign and were treated conservatively. However, five patients needed a percutaneous drainage of biloma, and one patient died consequently due to sepsis related to biliary fistula, a fact that highlights the importance of preventing biliary complications.

Similar to a concern after hepatectomy, liver dysfunction was observed in two patients from Group 1, both submitted to major resections. Similar to other series^4^ ^,^ ^12^ ^,^ ^27^, there was no statistical difference in the occurrence of hepatic dysfunction between first and re-hepatectomy patients (p=1). As this type of complication is intimately related to the amount of hepatic parenchyma resected and the volume of the liver remnant^21^ ^,^ ^26^, patients submitted to major resection are more prone to develop liver dysfunction.

Regarding the type of resection, minor hepatectomies were more prevalent in both groups (79% and 85.7% for Groups 1 and 2, respectively). However, we observed a tendency for more major hepatectomies in Group 1 (21% vs. 14.3%; p=0.606). Other studies^4^ ^,^ ^7^ ^,^ ^12^ ^,^ ^27^ also reported more major resection during first hepatectomy, while patients who had undergone re-hepatectomy also underwent more atypical and minor resections. This could be explained by the difficulties to perform major hepatectomies in patients previously submitted to surgery and chemotherapy, as well as to spare liver parenchyma in an organ already submitted to major parenchyma resection.

Four patients needed to be reoperated, three from Group 1, due to wound dehiscence and one from Group 2, due to choleperitonitis. From previous reports, bleeding and abdominal wall complications are the main indications for reoperation after hepatectomy^1^ ^,^ ^19^ ^,^ ^21^. We observed three deaths in the current study, two in Group 1 and one in Group 2, corresponding to a mortality rate of 1.4% and 3.6%, respectively (p=0.407), which is in line with the literature^4^ ^,^ ^12^ ^,^ ^27^.

## CONCLUSIONS

Repeated hepatic resections for CRLM became a safe procedure when performed by hepatobiliary teams with experience in complex liver resections. The results of the present study demonstrated no differences in morbidity or mortality between patients submitted to first and re-hepatectomies for CRLM, which reinforces that re-hepatectomy is an alternate option in the arsenal of treatments for these patients, with good outcomes and potentially cure possibilities.
